# The Gordian Knot of *C. auris*: If You Cannot Cut It, Prevent It

**DOI:** 10.3390/pathogens12121444

**Published:** 2023-12-13

**Authors:** Vasiliki Rapti, Katerina Iliopoulou, Garyfallia Poulakou

**Affiliations:** 1 Third Department of Internal Medicine, School of Medicine, National & Kapodistrian University of Athens, Sotiria General Hospital, 115 27 Athens, Greece; gpoulakou@gmail.com; 2 Lead Research Nurse, NHS Lothian, Edinburgh EH1 3EG, UK; catelios@yahoo.gr

**Keywords:** *Candida auris*, epidemiology, colonization, virulence, transmission, risk factors, outcomes, microbiological identification, infection control practices

## Abstract

Since its first description in 2009, *Candida auris* has, so far, resulted in large hospital outbreaks worldwide and is considered an emerging global public health threat. Exceptionally for yeast, it is gifted with a profoundly worrying invasive potential and high inter-patient transmissibility. At the same time, it is capable of colonizing and persisting in both patients and hospital settings for prolonged periods of time, thus creating a vicious cycle of acquisition, spreading, and infection. It exhibits various virulence qualities and thermotolerance, osmotolerance, filamentation, biofilm formation and hydrolytic enzyme production, which are mainly implicated in its pathogenesis. Owing to its unfavorable profile of resistance to diverse antifungal agents and the lack of effective treatment options, the implementation of robust infection prevention and control (IPC) practices is crucial for controlling and minimizing intra-hospital transmission of *C. auris*. Rapid and accurate microbiological identification, adherence to hand hygiene, use of adequate personal protective equipment (PPE), proper handling of catheters and implantable devices, contact isolation, periodical environmental decontamination, targeted screening, implementation of antimicrobial stewardship (AMS) programs and communication between healthcare facilities about residents’ *C. auris* colonization status are recognized as coherent strategies for preventing its spread. Current knowledge on *C. auris* epidemiology, clinical characteristics, and its mechanisms of pathogenicity are summarized in the present review and a comprehensive overview of IPC practices ensuring yeast prevention is also provided.

## 1. Introduction

In recent years, the global burden of invasive fungal infections (IFIs) has shown an upsurge, resulting from both the expansion of the immunocompromised population and the increase in invasive medical procedures [1]; thus, they embody a serious and growing public health threat. Approximately 70% of all IFIs reported annually are attributed to invasive candidiasis (IC), and *Candida* species have been identified as the predominant cause of nosocomial fungal infections, as well as the fourth leading cause of all hospital-acquired bloodstream infections (BSIs) [2,3]. Although *C. albicans* is the most commonly encountered pathogen among *Candida* species [2], we are most concerned about *C. auris*, a non-albicans species, which is classified as an urgent public health threat.

*C. auris* is a notorious cause of insidious hospital outbreaks and deep-seated infections [4,5]. It is primarily recovered from hospital environments [6,7,8], and, due to its unique traits, it is capable of colonizing and persisting in both patients and hospital settings for prolonged periods, thus creating a vicious cycle of acquisition, spreading, and infection, particularly in intensive care units (ICUs) [4,5,9]. Generally, it combines all the essential characteristics to be classified as an urgent public health threat [6,10], including the potentiality to spread rapidly through horizontal transmission, the ability to cause serious and life-threatening infections in susceptible individuals and the unfavorable profile of resistance to antifungal agents, alongside the lack of effective treatment options and standardized measures for prevention and control [4,5].

The aim of the present review is to summarize current knowledge on *C. auris* epidemiology, its clinical characteristics and mechanisms of pathogenicity, and provide a comprehensive overview of IPC measures and strategies.

## 2. Epidemiology

*C. auris* is a newly emerging multidrug-resistant yeast pathogen that has garnered the attention of the scientific and healthcare community. Whole-genome sequencing and phylogenetic analyses have revealed >4 major clades of *C. auris* with each one covering a distinct geographic area that emerged independently and nearly simultaneously at different locations across three continents [4,11,12,13].

Since its first description as a novel *Candida* species in 2009 in Japan, *C. auris* has, so far, been isolated in over 40 countries across six continents [4]. It is considered endemic in many regions of Africa and Asia [4,14,15,16], whereas prolonged and difficult-to-contain large-scale outbreaks, especially in ICUs, have been reported in Europe and the United States of America (USA) [4,5,17,18,19,20,21,22,23,24,25]. According to the European Centre for Disease Prevention and Control (ECDC), in a 4-year period (2013–2017), 620 *C. auris* cases were recorded in the European Union and European Economic Area, the vast majority of which derived from four large outbreaks in Spain and the United Kingdom [18,19,26,27,28]. Currently, the European burden of *C. auris* is steadily increasing; several sporadic cases have been observed all over Europe [26,29], and Spain turns out to be the only European country reporting regional endemicity [18]. In the USA, the Centers for Disease Control and Prevention (CDC) announced that clinical cases have increased each year since 2016, with the most rapid rise occurring during 2020–2021, and 17 states identified their first *C. auris* case during 2019–2021 [24,25].

The coronavirus disease 2019 (COVID-19) pandemic has further shaped the landscape of *C. auris* disease with a sharp rise in new cases of colonization and infection being observed [30,31], mainly attributed to the overload of the healthcare systems worldwide and the consequent compromised IPC practices [32,33,34], alongside the inability to implement adequate AMS programs. Concurrently, considering that critically ill COVID-19 patients tend to share risk factors, medications and underlying comorbidities with *C. auris*-infected patients [34], preventing the spread of this superbug in the ICU is challenging.

The prevalence and geographic extent of *C. auris* disease are possibly underestimated, mostly in low- and middle-income countries. The paucity of data arises from both the absence of a global identification strategy and the limited accuracy of available conventional diagnostic tools [35], since there is no widely used molecular method for rapid identification. Simultaneously, as a result of its low incidence, no large-scale epidemiology studies have been reported until now.

## 3. Colonization and Virulence Factors

The pathogenicity and virulence of *C. auris* are profoundly worrying, since it possesses its own unique characteristics that enhance invasive potential, favor antifungal tolerance and offer a growing advantage in natural and host niches. Thermotolerance, osmotolerance, filamentation, biofilm formation and hydrolytic enzyme production have been recognized as key components of *C. auris* pathogenesis.

Unlike most fungi that are unable to survive at human physiological temperatures, *C. auris* exhibits thermotolerance, allowing its growth at high temperatures, optimally at 37 °C, and maintaining viability up to 42 °C [4,36,37,38]. Of note, this new fungal disease was hypothesized to have originated from climate change, specifically global warming, based on phylogenetic analysis findings [39]. Another major trait is tolerance to osmotic stresses and high-saline environments (>10% NaCl, wt/vol) [4,36,37,38,40]. Notably, thermotolerance and osmotolerance, alongside limited susceptibility to commonly used disinfectants, are three cardinal characteristics of *C. auris* that permit its survival for days to weeks in diverse moist and dry surfaces [37,41,42,43,44]. It endures seven days on steel and porous surfaces [43], while on plastics, it persists for at least two weeks and can further survive in a-non culturable state for up to a month [37]. Consequently, persistence in harsh environmental conditions, a hallmark feature of *C. auris* that distinguishes it from the majority of other human fungal pathogens, leads to the observed high intra-hospital transmissibility and protracted outbreaks within healthcare settings [20,37,43].

Similar to other *Candida* species, it can undergo filamentation, a critical step in the fungal invasion of host tissues, both as pseudohyphae [28] and true hyphae [4]. Low temperatures were found to stimulate filamentous phenotype, whereas the human physiological temperature suppresses filamentous growth [45,46,47], leading to the conclusion that filamentous morphologies of *C. auris* may survive in the environment and on the host skin surface where the temperature is lower than inside the host. Additionally, a portion of *C. auris* isolates are capable of producing aggregates of pseudohyphal-like cells under high salt stress conditions or in biofilms [4,36,48]. The aggregating cells are known to display reduced virulence, but better survival capacity [28], selective tolerance to biocides and unique transferability to new, sterile surfaces after treatment [49,50].

Biofilm formation represents one of the main pathogenic traits. The majority of *C. auris* colonizing and clinical isolates exhibit an equal or even greater biofilm formation than *C. albicans* [48,51,52,53,54]. Singh and colleagues demonstrated that aggregated and non-aggregated phenotypes are predominantly associated with colonizing and clinical isolates, respectively, with the latter forming more robust biofilms [48]. Dense biofilms with up to 30-fold higher cellular burden than *C. albicans* are produced in contaminated with dried-up sweat and fatty acids surfaces, as might be the case during contact transmission from a colonized patient [51,55]. Besides, multilayer biofilms are rapidly formed in regions like the axilla and groin, while robust ones have been implicated in a variety of implant-associated infections, including BSIs and urinary tract infections (UTIs) [56,57]; hence, removal of central venous catheters (CVCs), other type of catheters and medical devices, where possible, should be advocated as an adjunctive treatment strategy [19,34]. Resistance to desiccation, osmotic stress [55,56] and various antifungals (e.g., fluconazole, voriconazole, echinocandins, amphotericin B) [51], as well as reduced susceptibility to potent skin disinfection agents, such as hydrogen peroxide and chlorhexidine [58], are other well-known characteristics of *C. auris* biofilms. Lastly, Kean and colleagues shed light on *C. auris* biofilm-mediated resistance [59]. During biofilm formation, *C. auris* induces the expression of a wide array of genes encoding cell-wall proteins and adhesins that favor biofilm adherence and persistence on biotic and abiotic surfaces. At the same time, genes encoding extracellular matrix proteins are upregulated and the produced matrix provides both structural integrity to the biofilm and yeast protection from environmental stressors, such as chemicals and disinfectants. Finally, a plethora of transporters and efflux-pumps are activated and resistance to antifungals and toxic chemicals is further promoted [59].

As far as virulence is concerned, *C. auris* expresses several virulence factors to degrade and invade host tissues [40,47]. Once in the environment, it activates the stress-activated protein kinase Hog1 for its adaptation to the dry abiotic milieu [55,56,60]. During this transition, phospholipases and proteinases are secreted in a strain-dependent manner and contribute to *C. auris* pathogenesis [52]. *C. auris* also stimulates hemolysin secretion to accelerate iron assimilation from the hemoglobin–heme group and eventually enhance its survival within the host [61,62].

## 4. Predisposing Factors for *C. auris* Infection, Clinical Spectrum and Outcomes

The predisposing risk factors for *C. auris* infection (Table 1) are similar to other *Candida* species, since they are opportunistic pathogens that primarily affect critically ill and immunocompromised patients [63].

Clinical manifestations of *C. auris* are diverse and range from colonization and mild, superficial skin infections to invasive disease and deep-seated infections [4,5]. Common sites of colonization include the skin, mostly the groin and axilla areas, rectum and mucosal surfaces of the urinary and respiratory tract (e.g., nares, and oropharynx) [9,15,19,30,41,70,75,76,77]. It is suggested that *C. auris* is incapable of colonizing anaerobic environments [5], like the gut, and the salivary antimicrobial peptide histatin 5 exerts a potent candidacidal effect on *C. auris* [71]. Therefore, unlike *C. albicans*, the colonization of the gastrointestinal tract is rare. Infection can occur at multiple body sites and *C. auris* has been isolated from both sterile (e.g., blood, cerebrospinal fluid, and bile) and non-sterile samples (e.g., urine, sputum, tissue, wound swabs, and catheter tips) [4,5,36,76]. Progression from colonization to invasive infections is estimated to occur in up to one fourth of affected patients [30,72], and candidemia is the predominant type of *C. auris* infection, followed by urinary tract, wound and ear infections, and rarely by respiratory tract or intra-abdominal infections, skin abscesses, myocarditis, meningitis and osteomyelitis [5]. It is noteworthy that *C. auris* candidemia usually follows colonization and multisite colonization is an independent risk factor for the development of candidemia [30]. Hence, the prompt identification of colonized patients at greater risk for developing candidemia may be beneficial for improving early diagnosis and preventing invasive infection through interventions on modifiable predictors. Lastly, the risk of infection of implantable devices (e.g., defibrillators, pacemakers, prosthetic joints, etc.) when the candidate is already colonized by *C. auris* has not yet been addressed in the literature, but according to the authors’ opinion, it is not negligible.

Invasive infections caused by *C. auris* are potentially life-threatening and increased mortality rates with significant geographic variation have been reported. In the literature, crude mortality ranges from 27% to 70% [11,30,64,65,66,68,70,73], whereas attributable mortality has not been adequately explored. Notably, a recent meta-analysis of 4733 *C. auris* cases, recorded from 2009 to 2019 in 33 countries worldwide, estimated a crude mortality of 39% and suggested a lower mortality in the European compared to the Asian continent (20% vs. 44%) [78]. Furthermore, as expected, BSIs incur a significant mortality toll, which can be as high as 70% [11,64,70,73], yet a crude mortality of 45% was documented in the aforementioned meta-analysis [78]. Additionally, crude 30-day mortality, reaching almost 60%, was revealed in case of recurrent candidemia in a study of 157 critically ill and *C. auris*-colonized patients, of whom 27 patients developed candidemia and 7 had a late recurrent episode [30]. This finding, however, should be interpreted with caution as it may reflect the severity of underlying noninfectious conditions in patients with prolonged ICU stay [79]. In co-infected COVID-19 patients, the estimated mortality is 44.4% and candidemia engenders a mortality of 64.7% [80]. Regarding the specific factors that are associated with unfavorable prognosis, advanced age, and presence of comorbidities, *C. auris* infection and particularly candidemia, as well as prolonged hospitalization, were identified in the survival analysis of a study that analyzed outcomes of 108 patients either infected or colonized by *C. auris* [66]. Similarly, in a retrospective analysis of 92 *C. auris*-affected patients, only candidemia was causally linked to greater mortality, while both infected and colonized cases shared comparable mortality [70].

## 5. Infection Prevention and Control Strategies

Prompt and accurate microbiological identification, as well as robust implementation of evidence-based IPC strategies are crucial for controlling and preventing *C. auris* outbreaks in healthcare settings. It is worth mentioning that *C. auris* is transmissible whether a patient is colonized or infected; thus, IPC measures are the same for both patient groups.

**Figure 1:**
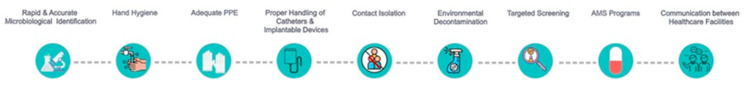
Optimal strategies for controlling and minimizing intra-hospital transmission of *C. auris.* AMS: antimicrobial stewardship; PPE: personal protective equipment.

The IPC strategies which have, so far, been successfully implemented in diverse healthcare settings worldwide [19,20,81,82,83,84,85,86,87,88,89] are illustrated in Figure 1 and they are discussed in the following paragraphs.

### 5.1. Rapid and Accurate Identification

*C. auris* has overlapping phenotypic characteristics with other closely related species, such as *C. haemulonii*, *C. sake* and *R. glutinis*, which compromise its rapid and accurate identification [90]. Due to misidentification issues, it is essential that microbiology laboratories update their commercial identification software to enable them to easily and efficiently identify *C. auris* cases [67], while the need for full identification in patients at greater risk for *C. auris* colonization or infection should be communicated.

A substantial progress has been made to improve *C. auris* identification methods. The first step included the development of a high-salt, high-temperature enrichment culture-based method that enables the accurate isolation of *C. auris* [37]. Once an isolate is obtained, matrix-assisted laser desorption/ionization time-of-flight (MALDI-TOF) mass spectrometry can be successfully applied for yeast identification, provided that the reference database contains the necessary information [6,91]. In case that MALDI-TOF is not available, sequence analysis of the internal transcribed spacer and D1/D2 region of the 28 s ribosomal deoxyribonucleic acid (DNA) can be performed [6,10]. Nevertheless, DNA sequencing is a time-consuming, expensive and is not available in all diagnostic lab methods, and its applicability may be limited, at least in developing countries [91]. For this reason, various sequencing-independent DNA-based methods, including end-point or multiplex polymerase chain reaction (PCR) assays, have been designed; they are highly sensitive and some of them were successfully validated for the direct detection of *C. auris* in clinical and environmental samples [91].

Lately, other culture-independent methods, such as PCR-restriction fragment length polymorphism (RFLP) [92], Taqman quantitative PCR (qPCR) [93,94], SYBR green qPCR [95], GPS™ MONODOSE CanAur dtec-qPCR (Genetic PCR Solutions™, Elche, Alicante, Spain) [96] and T2 Magnetic Resonance assay [97], have emerged as an attractive alternative approach for rapid detection, mostly in surveillance samples, as they are accompanied by accurate and reproducible identification of *C. auris* with a significantly reduced turnaround time compared to culture/MALDI-TOF-based methods.

### 5.2. Transmission

An alarming characteristic of *C. auris* is its inter-patient transmissibility and the fact that even colonized patients can serve as a reservoir for nosocomial spread. Specifically, it is efficiently transmitted from patient to patient, either directly or indirectly by sharing the same room or contaminated items, and by the colonized hands of healthcare workers (HCWs) [75]. Notably, contact with contaminated items is by far the most common method of colonization [20,37,43,44,64], and close contact of cases (e.g., current or past room contacts within a prior month) has a documented colonization rate of 12–21% [22,64]. The minimum contact period for the acquisition of *C. auris* from an infected person or surface is estimated to be 4 h [21], and invasive infections have occurred in patients within 48 h of ICU admission [98].

Sources of contamination have been found within the patient’s room, including bedding materials (e.g., bed rails and pans, mattress, linen, and pillows), furniture, door handles, flooring, walls, radiators, window sills, faucets and sinks [5,6,36,42,64,98,99]. It has also been isolated from high-touch surfaces and medical equipment, such as oxygen masks, axillary temperature probes, sphygmomanometer cuffs, pulse oxygen meters, electrocardiograph leads, catheter tips, infusion pumps and ventilators, particularly in outbreak settings [6,10,17,20,21,22,36,44,64,98,99,100]. For instance, following the identification of a cluster of *C. auris* infections in the neurosciences ICU, Eyre and colleagues concluded that patients exposed to reusable skin-surface axillary temperature probes had a sevenfold risk of infection or colonization [44].

#### Transmission-Based Precautions

In a systematic review of 17 studies reporting multidrug-resistant (MDR) outbreaks in ICUs, mainly caused by *C. auris* (n = 6), during the COVID-19 pandemic, the most commonly identified factors contributing to the outbreaks were inadequate PPE or a shortage of PPE, hand hygiene non-adherence, and high antibiotic use, followed by environmental contamination, prolonged critical illness and lack of trained HCWs [101]. Therefore, all HCWs attending *C. auris*-infected or -colonized patients should apply standard hand hygiene practices and perform adequate hand hygiene with soap and water, alcohol-based hand sanitizers, or chlorhexidine hand rubs [6,10,20,22,90,102,103,104]. Sharing of medical supplies and equipment is prohibited and use of disposable PPE (e.g., gloves, aprons, and gowns) is recommended [6,10,90,102,104]. Hospital infection control teams should raise awareness about *C. auris*, ensure that enough quantities of hand hygiene materials are available and monitor HCW adherence with recommended hand hygiene practices and PPE use, as well as train the personnel and retrain them at regular intervals. Additionally, as a low HCW/patient ratio is a well-established risk factor for MDR-organism (MDRO) transmission [105], a minimum number of HCWs should be designated for *C. auris* cases.

Strict isolation of patients harboring *C. auris* is recommended by the CDC and ECDC in order to prevent horizontal transfer to other patients [6,10]. Ideally, they should be placed in single-occupancy rooms with designated medical equipment and attached toilet facilities and be restricted there, except for medically necessary procedures [6,104]. Their rooms should be clearly marked and limited contact with visitors should be allowed. In case the number of single rooms is limited, they should be reserved for patients at the highest risk for transmission, such as those with uncontained secretions or diarrhea [90]. *C. auris* patients can also be cohorted [90,104], taking into account that these patients are usually co-infected with other MDROs. Strict isolation measures should not be an excuse for suboptimal patient care or result in the subject’s stigmatization [106]. Safety indicators and tools should be developed to avoid rupture in the flow of care as well as isolated patients’ emotional stress.

### 5.3. Decontamination and Disinfection Procedures

Extensive contamination of the healthcare environment has been described in facilities with *C. auris* outbreaks, highlighting the crucial role of enhanced daily and terminal disinfection in spread prevention [6,10]. Nevertheless, there are currently no standardized cleaning or disinfection procedures. Prior to decontamination, visible organic materials (e.g., body fluids) from the patient care area should be removed and cleaned [90], and the frequency of cleaning and disinfection is recommended to be at least twice daily, up to three times during outbreaks, and at least on all high-touch surfaces, such as bedrails and bedside tables [6]. Moreover, in case of patient discharge or transfer, terminal cleaning and disinfection should be carried out with great diligence and environmental sampling for *C. auris* culture should be performed in an outbreak setting [6,10,90,104]. To date, only sodium hypochlorite of 100 ppm concentration and topical hydrogen peroxide-based disinfectants are widely recommended for use [6,20,107,108], since commercially available products have been proven ineffective in eradicating *C. auris* [109]. For this reason, the US Environmental Protection Agency (EPA) has registered a list of qualified products for use and released a standardized quantitative disk carrier method, with the acronym SOP-MB-35-00, for evaluating the efficacy of antimicrobials against *C. auris* on hard, non-porous surfaces [110].

It is worth mentioning that disinfectant selection should be made weighting toxicity. For instance, exceptionally toxic disinfectants, like high-strength sodium hypochlorite agents of 5000 ppm concentration, should be reserved for terminal cleaning and not used on a regular basis. In addition to routine cleaning with disinfectants, peracetic acid [111], hydrogen peroxide < 1% [112], vaporized hydrogen peroxide [113], and ultraviolet subtype-C (UV-C) are other measures that can be used for optimal decontamination [114,115]. For example, UV-C is sufficient to prevent biofilm formation [115], and repeated flushing of colonized sinks in the patient’s room with ozonated water (2.5 ppm) (cycles of 30 s every 4 h) resulted in yeast elimination within 2 days [116]. Recently, silver nanoparticles are recognized as promising antifungal agents, as they exhibited both inhibitory effects on the growth of *C. auris* and antibiofilm formation activity [117]. Finally, as already mentioned, dedicated and single-use items (e.g., pillows, and bedding material) and equipment (e.g., thermometers, and blood pressure cuffs) should be used and, for equipment that cannot be dedicated to patients harboring *C. auris*, it is mandatory for it to be thoroughly disinfected after use [90,104].

### 5.4. Decolonization Protocols

The efficacy of decolonization protocols is still under investigation and not supported by regulatory bodies. Schelenz and colleagues suggest oral nystatin use, bathing with single-use wipes of 2% chlorhexidine gluconate twice daily and mouth washing with chlorhexidine 0.2% or chlorhexidine 1% dental gel in oropharyngeal-colonized, skin-colonized and ventilated patients, respectively [21]. However, if transient decolonization is achieved, the occurrence of recolonization is a potent scenario and high-touch areas may be the source of contamination where *C. auris* persists for long periods of time [20,21]. For this reason, patients with a history of colonization/infection by *C. auris* in the past should be considered as potentially colonized for at least one year in case of readmission, until surveillance cultures prove negative.

### 5.5. Targeted Screening and Labelling of the Patients

Targeted screening serves as a useful tool to prevent hospital transmission by rapidly implementing IPC practices. Once a *C. auris*-positive case has been identified, the infection control team should be immediately informed in order to trace the contact of origin and identify other potential patients who may have been exposed to the fungi. Moreover, *C. auris* cases should be followed until discharge and flagged for at least one year after the first negative screening culture [102], whereas HCWs and persons in close contact with them should be placed under strict contact precautions [21].

The CDC recommends that screening should be considered for close healthcare contacts with newly identified *C. auris* cases (colonized and/or infected) and patients reporting an overnight healthcare facility stay in a country outside the US in the previous year, especially if the country has documented *C. auris* cases [6]; pre-emptive screening of patients with international exposure is based on the finding that patients with a history of abroad hospitalization are at higher risk of MDRO carriage during ICU admission [118], and approximately 1 in 2 are estimated to be positive [119]. Notably, similar recommendations are supported by the ECDC [10].

Collection of one or more swabs of the patient’s axilla and groin regions is the screening method of choice [6], as they are thought to be consistent sites of colonization, albeit other body sites and specimens (e.g., nares, external ear canals, urine, wounds, and rectum) can be sampled, if indicated [10]. Initial screening and active surveillance may be conducted with one of the rapid microbial-detection methods mentioned above, followed by culture and molecular typing for epidemiological investigation. In the literature, there are several paradigms of successful screening programs [109,120,121], hence emphasizing the need and utility of a rapid and automated molecular surveillance admission screening, primarily in endemic regions.

### 5.6. Handling of Catheters and Implantable Devices

In the context of infection source control, urinary catheter or CVC removal in *C. auris*-infected patients is crucial [17,19,122,123,124]. Strict adherence to central, peripheral, and urinary catheter care bundles and proper care of the tracheostomy site are essential preventive measures [6], and placing chlorhexidine-impregnated protective disks in all CVC exit sites may result in the reduction of central line-associated BSIs [21]. As previously discussed, the risk of infection of implantable devices in *C. auris* colonized patients does not seem negligible. Therefore, although there are no specific recommendations to our knowledge, we would suggest rigorous skin preparation before the implantation and a single dose of an antifungal agent preoperatively, according to the antibiogram, prioritizing agents acting in the presence of biofilm.

### 5.7. AMS Programs Implementation

Antifungal stewardship (AFS) programs are recognized as an essential tool for minimizing antifungal overuse or misuse [122,123], and *C. auris* emergence provided the impetus for their broad implementation. For instance, in the past, AFS was less attractive and in a survey of AFS initiatives in English acute hospitals, only a minority (11%) conducted a dedicated AFS program [125]. Considering that antifungal drug classes are limited compared with antibacterial classes and the fact that *C. auris* presents reduced susceptibility to azoles, polyenes, and echinocandins, in a clade-dependent manner [126], judicious use of antifungals is necessary. Therefore, intensification of AFS within the AMS programs is of great importance. Decreasing empiric antifungal treatment as much as possible and performing de-escalation that includes antifungal agents are important elements of AFS programs, along with curtailing unnecessary prolongation of antifungal courses. Specific training and feedback of all stakeholders is warranted, as in the case of antibiotic stewardship strategies.

Prior or continual exposure to broad-spectrum antimicrobial therapy, a well-established risk factor for *C. auris* acquisition, has a long-lasting effect on skin microbiota [125]. Interestingly, *C. auris*-positive individuals were shown to harbor different commensal bacteria and fungi communities [126], although it should be clarified whether dysbiosis contributes to *C. auris* colonization or *C. auris* colonization promotes the alteration of microbial communities. Therefore, the conduction of studies investigating the intersection of skin microbiota and *C. auris* skin colonization is needed.

## 6. Conclusions

*C. auris* is considered an emerging health threat with reports of both sporadic cases and outbreaks in healthcare facilities from diverse countries across six continents. It is implicated in a variety of invasive, potentially life-threatening infections and the patients most commonly affected include the elderly with debilitating comorbidities, catheters or indwelling medical devices, and prolonged ICU stay that have been exposed to broad-spectrum antimicrobial agents, administered parenteral nutrition or recently undergone invasive medical procedures. Faulty and delayed detection by conventional diagnostic tools, high inter-patient transmissibility, intrinsic or acquired resistance to one or more antifungal drugs, and limited susceptibility to commonly used disinfectants are the main reasons hindering the adequate management of *C. auris* outbreaks. Hence, establishing a multidisciplinary model and bundling of practices for controlling and preventing its spread are of utmost importance.

## Figures and Tables

**Table 1:** Predisposing factors for *C. auris* infection.

Predisposing Factors	Reference
Chronic Disease (e.g., cardiovascular, respiratory and renal disease, diabetes mellitus)	[4,11,17,30,64,65,66,67,68]
Immunosuppression (e.g., malignancy, AIDS, organ transplantation, immunosuppressive agents)	[4,11,30,64,65,67,69]
Catheters and Indwelling Medical Devices	[4,11,17,30,41,64,65,66,67,70,71,72,73]
Mechanical Ventilation	[4,30,64,66,70,73]
Prolonged Hospital and ICU stay	[30,65,67,70]
Broad-spectrum Antimicrobial *and *Antifungal Therapy	[11,19,30,64,65,66,67,68,70,73]
Parenteral Nutrition	[19,30,65]
Recently Performed Invasive Medical Procedures (e.g., surgery)	[11,19,30,65,67,68,70]
Age (e.g., preterm infants and elderly)	[65,67]
Male sex and country-specific health factors	[65,67]
Diarrhea	[74]
Tetracyclines consumption (minocycline and tigecycline)	[74]

AIDS: Acquired immunodeficiency syndrome; ICU: Intensive care unit.

## Data Availability

Not applicable.
